# Mechanical properties and formulation of hydrophilic fiber and shrimp shell combination as a novel eco-friendly dental restoration material

**DOI:** 10.1016/j.heliyon.2024.e34180

**Published:** 2024-07-05

**Authors:** Dewi Nur Fadhila, Alisha Zafirah Ridwan, Nur Aqilah Amir, Andi Abdillah, Ratih Kartini N, Hasanuddin H, Nursyamsi Djamaluddin

**Affiliations:** a Dental Education Study Program, Faculty of Dentistry, Hasanuddin University, Indonesia; b Pharmacy Study Program, Faculty of Pharmacy, Hasanuddin University, Indonesia; c Department of Dental Public Health and Preventive Dentistry, Faculty of Dentistry, Hasanuddin University, Indonesia

**Keywords:** *Chitosan*, *Eco-friendly*, *Composite resin*, *Cellulose*

## Abstract

This study evaluates the mechanical properties and formulation of dental restoration material comprised of cellulose acetate (CA) from water hyacinth and chitosan (C) from white shrimp shells. The research phases included extraction, formulation, functional group testing, antibacterial, toxicity, water absorption and solubility, compressive, shear, tensile, hardness, microleakage, thermal expansion, and shrinkage. The experimental data were analyzed using probit regression, one-way ANOVA, and Kruskal-Wallis test. The data showed that CA and C had microxyl and amine groups, could inhibit *S. mutans*, and were non-toxic. Composite resins were divided into nine formulations with different concentrations: F1 (1 % CA + 3 % C), F2 (1 % CA + 5 % C), F3 (1 % CA + 7 % C), F4 (3 % CA + 3 % C), F5 (3 % CA + 5 % C), F6 (3 % CA + 7 % C), F7 (5 % CA + 3 % C), F8 (5 % CA + 5 % C), and F9 (5 % CA + 7 % C). The F9 has mechanical strength close to the control group, with 113.33 μg/mm^3^ absorption, 80 μg/mm^3^ solubility, 32.67 Mpa compressive strength, 17.18 Mpa tensile strength, and no shrinkage. It shows that F9 has potential as an eco-friendly dental filling material. The present study completed the development of a formulation for a restoration material by combining water hyacinth fiber and shrimp skin chitosan. The outcomes of a comparative analysis of the mechanical properties of synthetic composite resins and water hyacinth fiber composites containing shrimp skin chitosan revealed that the F9 formulation (CA 5 % + C 7 %) exhibited the following fiber: absorption, compressive strength, tensile strength, hardness, and thermal expansion.

## 1 Introduction

Caries is a global oral health problem. About 2.4 billion (36 %) of the world's population have caries in permanent teeth, and more than 530 million children lose their primary teeth due to caries [1]. The prevalence rate of caries in Indonesia reached 88.8 % in 2018 [2]. Untreated caries affects a patient's quality of life because they cause pain, chewing problems, stunting, and malocclusion and may affect systemic conditions [3]. The most popular caries treatment solution is composite resin fillings due to their excellent aesthetics, mechanical properties, durability, biocompatibility, non-toxicity, and practical manipulation [4,5]. However, composite resins are non-biodegradable due to polymerization shrinkage, high water absorption, and solubility; this results in a reduction in mechanical strength and, if the monomer conversion is incomplete like BisGMA, the possibility of human exposure to the bisphenol-A compound, which is known to be responsible to increase systemic health risks such as male reproductive abnormalities [[6], [7], [8], [9]]. Composite resin materials consist of an organic resin matrix, inorganic and organosilane fillers (coupling agents), and additional components such as activators, pigments, inhibitors, and ultraviolet absorbents [7]. Fillers are essential in shaping mechanical properties such as modulus, strength, fracture toughness, fatigue life, hardness, and wear resistance [9,10]. Unfortunately, composite resin materials are nonrenewable, so more environmentally friendly materials should be proposed.

Cellulose can be an alternative to filler replacement in composite resins due to its exceptional mechanical strength [11]. Water hyacinth (*Eichhornia crassipes*) is one of the natural compounds that can be used as cellulose strands, although its highly aggressive invasive species has emerged as a significant concern and poses a threat to aquatic ecosystems in over fifty countries [12]. Water hyacinth is a weed characterized by its rapid growth and capacity to alter water salinity, reduce dissolved oxygen levels, and induce water shallowing [13]. Due to its substantial cellulose content (66.87 %), water hyacinth's resistance to water and degradation, and limited application history shows potential as a reinforcing filler for composite resin [14,15]. The previous study showed that this polymer is non-toxic, biodegradable, and biocompatible, has a high affinity for other substances, and has excellent mechanical and regenerative properties [16]. Nevertheless, the hydrophilic nature of cellulose in water hyacinth necessitates a modification by acetylating the hydroxyl group; this process transforms cellulose into cellulose acetate [17]. Cellulose acetate has low water absorption and is resistant to high temperatures [11].

In the context of composite resins, the water absorption capacity of the matrix is enhanced due to the absorbent properties of *bisphenol a glycidyl methacrylate* (BisGMA), *urethane dimethacrylate* (UDMA), and *triethylene glycol dimethacrylate* (TEGDMA) [18]. Because it absorbs water at a physiological pH of seven, such as in the oral cavity, chitosan can be utilized as an alternative to matrix material [19]. Chitosan, formed through the N-deacetylation of chitin, is an inherent biopolymer known as poly-*J*-1,4-glucosamine. As evidenced by a degree of deacetylation (DD) exceeding 90 %, chitosan derived from shrimp (Crustacea) is the most effective material-enhancing agent. With a degree of deacetylation of 92 %, chitosan derived from the shell of the white shrimp species *Litopenaeus vannamei* contains 42.2 % chitosan. The previous study showed that incorporating chitosan into composite resins enhances their adhesion to materials and biocompatibility. It has better antibacterial properties than the composite resin without adding chitosan and without changing the composite's flexural strength and mechanical properties [20].

This study aims to develop an advanced and ecologically sustainable dental restoration material formulation by combining chitosan derived from shrimp skin and cellulose acetate derived from water hyacinth as a matrix and filler, respectively. The formulation is designed to address tooth decay and environmental issues in alignment with the sustainable development goals, with a particular focus on goal 3, which pertains to promoting a healthy and prosperous life, and goal 15, which supports the management and preservation of biodiversity.

## 2 Materials and methods

### 2.1 Location and time

This experimental research uses a group randomized design method with three replications between July 3 and September 30, 2023, in eight laboratories at Hasanuddin University and Ujung Pandang State Polytechnic, Makassar-Indonesia. Water hyacinth was obtained from Hasanuddin University Lake, while shrimp shells were purchased from PT Bomar. The research ethic was approved by the Health Research Ethics Committee of the Faculty of Dentistry Hasanuddin University and Dental Hospital of Hasanuddin University number 0120/PL.09/KEPK FKG-RSGM UNHAS/2023 on June 23, 2023.

### 2.2 Materials and tool

A number of materials were utilized in this laboratory research. This includes aluminum foil, distilled water, acetic anhydride, a blue tip, a Petri dish, CH3COOH glacial, DMSO, iodine salt, a measuring cup, H2SO4, HCl, pH paper, filter paper, Artemia salina shrimp larvae, methanol, methylene blue, sodium agar, NaOCl, NaOH, paper disk, polyvinyl alcohol (PVA), and a sterile swab. The control group used composite nanofiller resin (CN). An analytical balance used Fourier Transform Infrared (FTIR), Universal Testing Machine (UTM), stirring rod, Petri dish, Buchner funnel, measuring cup, hot plate, incubator, Erlenmeyer flask, volumetric flask, laminar air flow, magnetic stirrer, oven, drop pipette, vacuum pump, horn spoon, spatula, and analytical balance (HVN).

### 2.3 Water hyacinth extraction

Sorting the stems of water hyacinth was the initial step; they were subsequently washed, cut, dried, mashed, and stored in a container [21]. Then, a 15 g sample was homogenized at 70–80 °C for 2 h after being extracted with 4 % NaOH solvent, and 3 % NaOCl was used to bleach the sample at 70–80 °C for 3 h. Following filtration, the remaining substance was dried at 105 °C for 2 h [22]. Four stages comprise the acetylation process, which reduces the hydrophilic nature of cellulose: activation, acetylation, hydrolysis, and purification. To initiate the activation process, 10 g of cellulose was dissolved in a 100 ml solution containing 30 % CH3COOH glacial. The mixture was stirred continuously at 200 rpm for 1 h. Following this, 50 ml of acetic anhydride and 0.5 ml of sulfuric acid catalyst are added with a constant stirring at 200 rpm for 1.5 h at 40 °C. Following this, 25 ml of distilled water and 50 ml of CH3COOH glacial, both heated to 50 °C with constant stirring at 250 rpm for 30 min, were added to initiate the hydrolysis process. Further, the solution was distilled and purified with water for 15 min, or until the pH was neutral and the CH3COOH odor had vanished, at which point it was precipitated with methanol solution. Following filtration, the precipitate was dried for 6 h at 55 °C in an oven [23].

### 2.4 Shrimp shell extraction

Deacetylation of chitin into chitosan, deproteinization, and demineralization comprise the three steps involved in chitosan extraction [24]. Shrimp shell powder was dissolved in a 4 % NaOH solution at 1:10 (g/ml) to accomplish deproteinization. The solution was then heated at 70 °C for 2 h before being filtered and rinsed with distilled water until the pH was neutral. The formed solid was cooled, dried for 6 h at 80 °C in an oven, and weighed. After continuing the demineralization process, the protein-free powder was dissolved in a 1.5 M HCl solution at a 1:15 (g/ml) ratio while being stirred for 1 h at room temperature. The resulting solution was filtered and rinsed with distilled water until the pH reached neutral. Subsequently, dry chitin powder was dissolved in a 1:10 (g/ml) ratio of 50 % NaOH solution in distilled water and filtered. The solution was then heated at 90 °C for 2 h before being rinsed with distilled water. It was weighed after pouring the solid into a porcelain cup and drying it for 6 h at 80 °C in an oven [25].

### 2.5 FTIR analysis

The Cellulose Acetate and Chitosan were placed on Attenuated Total Reflectance (ATR) plate at a controlled ambient temperature (25 °C) and scanned using an FTIR spectrophotometer (ABB MN3000, Clairet Scientific, Northampton, UK) at a wavelength of 4000-500 cm^−1^ equipped with a deuterated triglycine sulfate (DTGS) detector and potassium bromide (KBr) as the beam splitter, recorded for 32 scans at 8 cm^−1^ resolution. These spectra were recorded as absorbance values at each data point in triplicate [26].

### 2.6 Antibacterial test

Flying Paper Disk (FPD) testing was employed to conduct antibacterial analysis using *Streptococcus mutans* bacteria [27]. A 24-h incubation period of 37 °C was applied to a pure culture of *Streptococcus mutans* bacteria. After suspending the isolate in 2 ml of a 0.9 % NaCl solution, a sterile swab was used to evenly scratch the surface of the NA media [28]. A 50 L dripping of the acetic acid-dissolved extract onto a paper disk was followed by its evaporation and subsequent application to the agar surface. A positive control is impregnated with 30 μg amoxicillin, whereas the negative control solely comprises a solvent.

### 2.7 Toxicity test

In the present study, the toxicity test was carried out using the *Brine Shrimp Lethality* Test (BSLT). BSLT was performed to study the toxicity with *Artemia salina* larvae. The toxicity of the Cellulose acetate and Chitosan mixture was tested at concentrations of 62.5, 125, 250, 500, and 1000 ppm in 10 ml seawater solution and 0 ppm without the test substance as a control, which was added with 1 % DMSO solvent (v/v). Then 30 Artemia salina Leach shrimp larvae aged 48 h were used at each concentration tested. The toxic effect was obtained from observations by calculating the percentage of dead larvae at each concentration within 24 h for each replication (three replications were used for each concentration) are tallied utilizing the following equation:

Eq. (A.1) $$\%\;\text{Mortality}=\frac{\text{Total}\;\text{death}\;\text{larvae}}{\text{Total}\;\text{larvae}}$$

Subsequently, the data set underwent statistical analysis via probit regression, employing a 95 % confidence level [29]. The results were analyzed using probit analysis so that the LC50 value was obtained using the Software Package used for Statistical Analysis (Version 26.0; SPSS Inc., Chicago, IL, USA).

### 2.8 Formulation preparation

After the extraction of cellulose acetate from water hyacinth and chitosan from shrimp skin (Table 1), the next step is to make a composite resin formulation by mixing 2 g of PVA with 10 ml of distilled water at 80 °C. When it has thickened, chitosan (C) (3 %, 5 %, 7 % with w/v formula) is dissolved with 5 ml of 1 % CH3COOH and then added with cellulose acetate (CA) (1 %, 3 %, 5 % with w/v formula). The three ingredients were then mixed using a hot plate at 100 °C and then molded on a Petri dish and waited to harden. Cellulose acetate (CA) with concentrations of 1 %, 3 %, and 5 % was combined with chitosan (C) with concentrations of 3 %, 5 %, and 7 % using the group randomized design method to obtain the formulations. In the formulation, PVA was added as a coupling agent and 1 % (v/v) CH3COOH as a chitosan solvent.

**Table 1:** Nine formulations were derived from the findings of this study.

Formulation	F1	F2	F3	F4	F5	F6	F7	F8	F9
Cellulose Acetate	1 % (0.15 g)	1 % (0.15 g)	1 % (0.15 g)	3 % (0.45 g)	3 % (0.45 g)	3 % (0.45 g)	5 % (0.75 g)	5 % (0.75 g)	5 % (0,75 g)
Chitosan	3 % (0.45 g)	5 % (0.75 g)	7 % (1.05 g)	3 % (0.45 g)	5 % (0.75 g)	7 % (1.05 g)	3 % (0.45 g)	5 % (0.75 g)	7 % (1.05 g)
Pva	2 g	2 g	2 g	2 g	2 g	2 g	2 g	2 g	2 g
Aquades	10 ml	10 ml	10 ml	10 ml	10 ml	10 ml	10 ml	10 ml	10 ml
CH3COOH	5 ml	5 ml	5 ml	5 ml	5 ml	5 ml	5 ml	5 ml	5 ml

### 2.9 Absorption and solubility test

The sample was imprinted onto a mold measuring 25 mm in length, 2 mm in width, and 1 mm in height. The volume of the sample was determined by utilizing a digital caliper and the subsequent formula:

Eq. (A.2) V=l×w×h

The variables l, w, and h represent length, width, and height, respectively. Immersion was conducted for one day using 2,5 ml of saliva per sample. The sample was drained for 15 min after a one-day immersion period before absorption measurement, which was conducted utilizing the aforementioned formula:

Eq. (A.3) $$\text{Sp}=\frac{m2‐m3}{V}$$

The symbols Sp (Sorption or absorption), m2 (mass after soaking in μg), m3 (mass after drying in μg), and V (volume of the sample) are denoted (mm^3^). The solubility of composite resins is determined by utilizing samples that have undergone absorption testing. After one day of drying on silica gel, the samples were placed in a desiccator. The mass of the dried samples was determined by weighing them on an analytical balance. The following formula was used to determine solubility:

Eq. (A.4) $$\text{SL}=\frac{m1‐m3}{V}$$

SL represents solubility; m1 denotes initial mass in μg; m3 signifies mass in μg after drying; and V signifies sample volume in mm^3^ [30].

### 2.10 Micro-leakage test

The sample was imprinted onto a mold measuring 2 mm in height and 5 mm in diameter. Then, 1 ml of methylene blue was drip-treated onto the sample and left undisturbed for 24 h. The sample was divided into equal portions in half. The specimen underwent examination through the measurement of the penetration rate of methylene blue. The dye penetration metric was utilized to assess the test outcomes. The dye penetration score is classified into three categories: score 0 (zero) indicates the absence of dye solution penetration; score 1 (one) signifies dye solution penetration on half of the surface; and score 2 (two) indicates dye solution penetration on more than half of the entire surface [31].

### 2.11 Compressive strength test

A UTM was employed to perform compressive strength tests. The load was applied at a crosshead speed of 1 mm/min while the specimen was affixed to the lower jaw of the UTM; the specimen remained broken throughout. Using the following formula, the compressive strength (Mpa) was determined:

Eq. (A.5) $$\text{CS}=\frac{F}{\text{Area}\;\text{of}\;a\;\text{cross}‐\text{section}}$$

The maximum breaking load, denoted in newtons, is F. For the calculation of the cross-sectional area, see Equation (A.2) [32].

### 2.12 Shear strength test

UTM-based shear strength testing. The shear strength of a material refers to its highest capacity to endure loads that induce shear deformation within the material prior to its release [33].

### 2.13 Tensile strength test

Evaluation of tensile strength using UTM. The material is subjected to the tensile test by laying it on top of the UTM until it is severed. The value and maximum strength of the material will then be displayed via parameter value display [34].

### 2.14 Hardness test

Hardness test with a tool using HVN. In the Vickers method test, an indenter is used as a small pyramid to provide a load on the material's surface. The test sample and the results obtained are entered into the formula [35].

### 2.15 Thermal expansion and shrinkage test

An oven was preheated to 250 °C [36] to conduct thermal expansion and shrinkage tests.

### 2.16 Data collection and analysis

The statistical analysis was conducted using SPSS version 26, employing probit regression, one-way ANOVA, and Kruskal-Wallis. The interpretation was provided as a narrative supplemented by tables, graphs, and figures. The deductive approach was employed to infer conclusions from the research findings.

## 3 Results

### **3.1** Functional group test

**Fig. 1:**
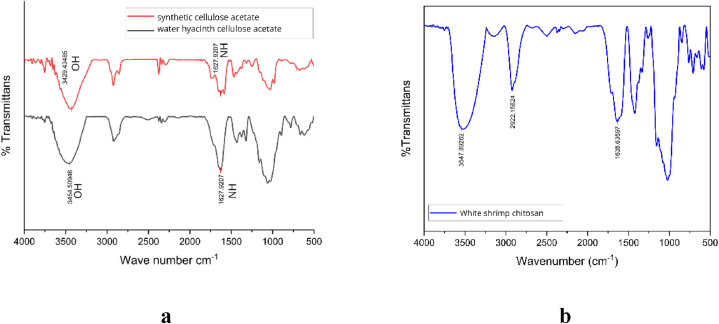
a. FTIR spectrum of cellulose acetate, b. FTIR spectrum of chitosan.

Fig. 1a and b present the functional group test results of synthetic cellulose acetate, water hyacinth cellulose acetate, and white shrimp chitosan using FTIR.

The peak wavelength of 3454 cm^−1^ observed in CA water hyacinth is longer than that of synthetic cellulose, as shown in Fig. 1a. The findings fall within the wavelength at 3570 - 3200 cm-1, suggesting hydroxyl groups (-OH) exist. Furthermore, the peak wave number for CA water hyacinth and CA synthesis was 1627 cm^−1^. The number, which falls between 1650 and 1590 cm^−1^, signifies the existence of primary amine groups (NH). CA primarily comprises hydroxyl groups and primary amines as functional groups [36]. The FTIR analysis of C from white shrimp yielded a peak at wavelength 3527 cm^−1^, as illustrated in Fig. 1b. The obtained results fall within the frequency range of 3200–3600 cm^−1^, which corresponds to the hydroxyl group (-OH); 1635 cm^−1^, which corresponds to the primary amine group (NH); and 2922 cm^−1^, which falls within the frequency range of 2850–3000 cm^−1^, signifying the existence of methylene groups (>CH). the presence of bent methylene groups (>CH2) at 1423 cm^−1^ in the range of 1350–1480 cm^−1^, and phenol (C–O) groups at 1155 cm^−1^ in the range of 1000–1300 cm^−1^ [37]. These results demonstrate the suitability of the functional groups present in chitosan.

### 3.2 Antibacterial test

**Fig. 2:**
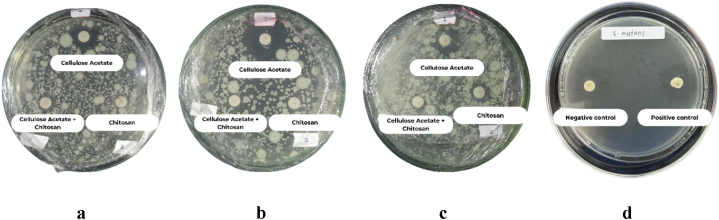
Growth inhibition zone of *S. mutans* bacteria. a.Replication 1, b. Replication 2, c. Replication 3, d. control (−) and (+).

The findings depicted in Fig. 2(a–c) indicate that the cellulose acetate, chitosan, and combined cellulose samples exhibit the capacity to impede the growth of *Streptococcus mutans.* In contrast, Fig. 2d shows the positive and negative control (amoxicillin). This is confirmed by the clear zone areas [27] shown in Table 2 at 12,80 mm, 8,70 mm, and 7,57 mm, respectively. This demonstrates that dental fillings containing the CA + C mixture could possess antibacterial properties against *Streptococcus mutans* bacteria.

**Table 2:** Inhibition zone diameters of samples against *S. mutans* growth.

Sample	Replication	Mean zone of inhibition (mm)
Cellulose	3	12,800
Chitosan	3	8700
Cellulose + Chitosan	3	7566
Negative control	1	0
Positive control	1	20,100

### 3.3 Toxicity test

**Fig. 3:**
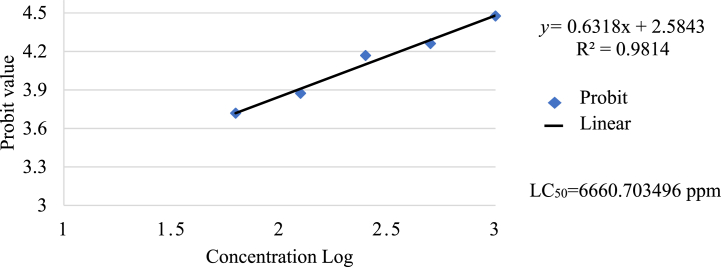
Correlation between the concentration of a cellulose acetate and chitosan mixture dissolved in DMSO solvent and Artemia Salina larvae's mortality percentage (probit value).

The data presented in Table 3 indicates that the highest concentration, at 1000 ppm, showed the highest larva mortality of 30 %, while the lowest concentration, at 62.5 ppm, showed larva mortality of 10 %. However, probit analysis of the CA + C, as shown in Fig. 3, depicted that the LC50 value was 6660.703496 ppm (>1000 ppm), which showed that the CA and C mixture exhibits no toxicity, rendering it a viable and secure substitute for dental fillings. These findings are consistent with previous research [38] that suggests bioactive components can be beneficial when administered in low doses but are toxic when taken in high doses.

**Table 3:** Concentration of cellulose acetate and chitosan mixture on the number of *Artemia salina* larvae mortality.

Concentration (ppm)	Replication			Total deaths	Percent mortality	Total artemia
	1	2	3			
0	0	0	0	0	0 %	30
62.5	1	1	1	3	10 %	30
125	1	2	1	4	13 %	30
250	3	3	0	6	20 %	30
500	2	4	1	7	23 %	30
1000	4	2	3	9	30 %	30

### 3.4 Absorption and solubility test

**Fig. 4:**
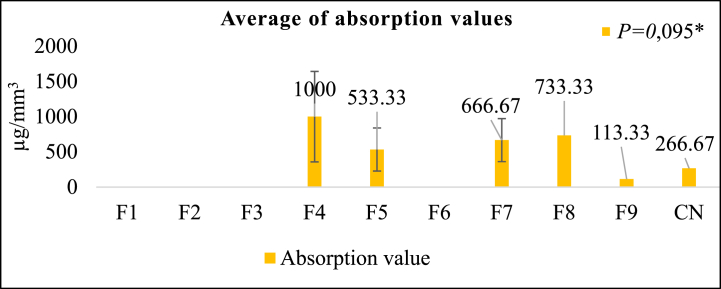
Absorption test results (μg/mm^3^) *Kruskal-Wallis test*,* Significance *P > 0*.05.

**Fig. 5:**
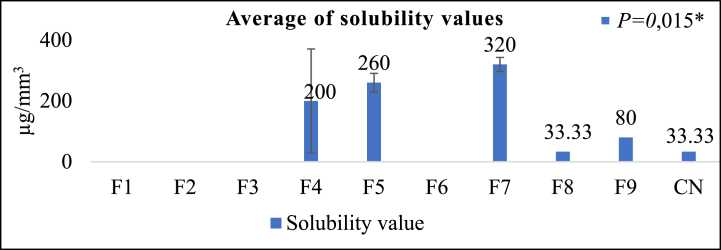
Solubility test results (μg/mm^3^) *Kruskal-Wallis test*,* Significance *P < 0*.05.

Fig. 4 illustrates that formulation F9 exhibits the lowest absorption test value of 113.33 ± 11.55 μg/mm^3^. Subsequently, F5, F7, F8, and F4 follow suit. Furthermore, at 33.33 ± 30.55 μg/mm^3^, F8 exhibits the least solubility value, which F9, F4, F5, and F7 succeed. F9 exhibited a reduced absorption value and solubility of F8 compared to the control group, demonstrating a solubility of 33.33 μg/mm^3^ and an absorption value of 266.67 μg/mm^3^. The results of the Kruskal-Wallis test indicated that the absorption test yielded no statistically significant difference, whereas the solubility test (Fig. 5) revealed a significant difference. An excessive degree of water absorption and solubility may result in a deterioration of the mechanical properties of the composite resin, thereby compromising its long-term durability [39]. The absorption test outcomes indicated that samples F4, F5, F7, F8, and F9 did not undergo significant absorption. In contrast, samples F1, F2, F3, and F6 underwent substantial absorption; consequently, additional tests were not feasible.

### 3.5 Compressive strength test

**Fig. 6:**
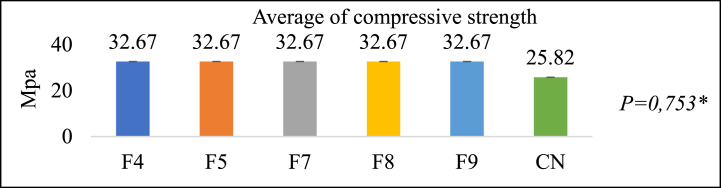
Compressive test results (Mpa) *One Way ANOVA Test Significance *P > 0*.05.

Fig. 6 illustrates that the compressive strength of all test groups is significantly higher at 32.67 ± 0.753 Mpa compared to the control group's 25.82 Mpa. According to the One-Way ANOVA test results, no significant difference exists. The increase in filler content influences the increase in mechanical strength [10]. As the compressive strength of a material increases, so do its strength and resistance to wear [40].

### 3.6 Shear strength test

**Fig. 7:**
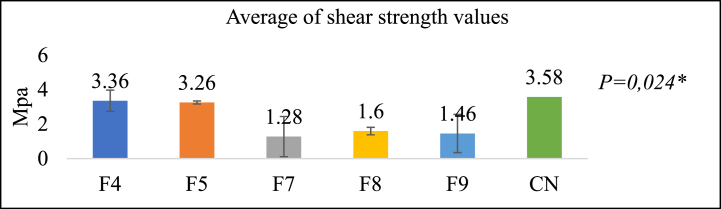
Shear test results (Mpa) *One Way Annova test*,* Significance *P < 0*.05.

Fig. 7 illustrates the outcomes of the shear strength tests, which indicate that group F4 consists of the formulation with the most significant value (3.36 ± 1.34 Mpa), followed by F5, F8, F9, and F7. The 3.58 Mpa is a shear strength value comparable to that of the control group for F4. The One-Way ANOVA test results indicate the presence of a statistically significant difference. An improvement in the bond with the tooth is directly proportional to the shear strength value [8].

### 3.7 Tensile strength test

**Fig. 8:**
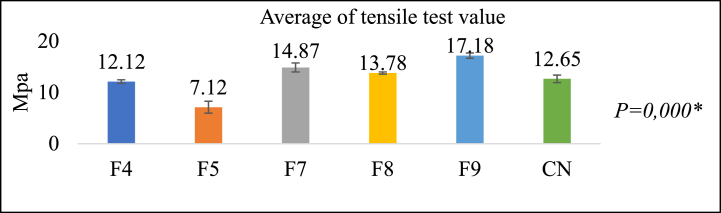
Tensile test results (Mpa) *One Way ANOVA test*,* Significance *P < 0*.05.

As illustrated in Fig. 8, the formulation denoted as group F9 exhibited the highest tensile strength value of 17.18 ± 0.51 Mpa. Subsequently, F7, F8, F4, and F5 recorded lower values. F9 exhibits a 12.65 Mpa increase in tensile strength relative to the control team. Significant variation exists, as indicated by the outcomes of the One-Way ANOVA test. A formulation will resist tensile loads more as its tensile strength value increases [41].

### 3.8 Hardness test

**Fig. 9:**
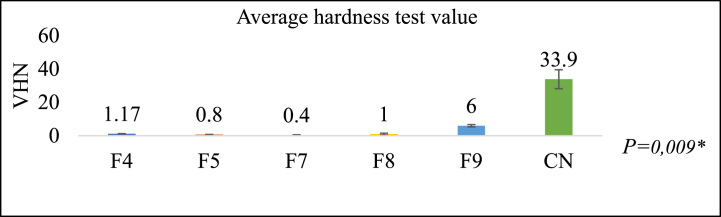
Hardness test results (VHN) *Kruskal-Wallis test*,* Significance *P < 0*.05.

Fig. 9 illustrates the hardness test outcomes, wherein group F9 comprises the formulation with the highest recorded value of 6.00 ± 0.69 VHN. Subsequently, F4, F8, F5, and F7 follow suit. At 33.9 VHN, the hardness value of F9 is lower than that of the control group. The Kruskal-Wallis test indicates the presence of a statistically significant difference. A material with a high hardness value will be more resistant to abrasion and scratches, preventing it from deforming easily under various forces but becoming easily broken or brittle [42].

### 3.9 Micro leakage test

**Fig. 10:**
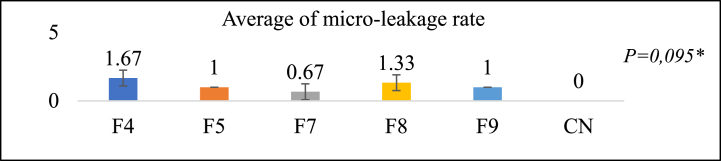
Micro-leakage test results *Kruskal-Wallis test*,* Significance *P > 0*.05.

Fig. 10 illustrates the microleakage test outcomes, revealing that the F7 group comprises the formulation with the least significant value (±0.57 for 0.67), followed by F9, F5, F8, and F4. F7 exhibits a microleakage value close to the control group's zero value while remaining within the 0.5–2.6 range [43]. As determined by the Kruskal-Wallis test, no statistically significant difference existed. Microleakage occurs when the restoration material fails to adequately adapt to the tooth cavity's wall surface, resulting in a gap formation [31]. This gap can facilitate the ingress of bacteria and fluids.

### 3.10 Thermal expansion and shrinkage test

**Fig. 11:**
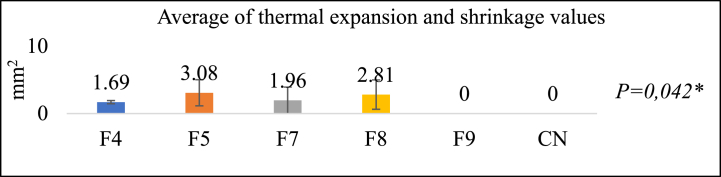
Thermal expansion and shrinkage test results *Kruskal-Wallis test*,* Significance *P < 0*.05.

Fig. 11 illustrates the outcomes of the thermal expansion and shrinkage test. Among the formulations examined, group F9 exhibits the least shrinkage (0 units), with groups F4, F7, F8, and F5. F9 shows the same reduction in size as the control group, which does not undergo any size reduction. A significant distinction can be observed, as indicated by the Kruskal-Wallis test results. Shrinkage-induced alterations in the strengths of composite resins contrast with the attachment strength, thereby impeding the resins' ability to adhere to the tooth's surface [43]. Microcracks can induce patch failure when subjected to high masticatory force and thermal stress [44].

## 4 Discussion

The present study evaluated the mechanical properties and formulation of a dental restoration material composed of chitosan (C) extracted from white shrimp shells and cellulose acetate (CA) derived from water hyacinth. The study employed a series of randomized laboratory experiments, replicating the process three times at Hasanuddin University and Ujung Pandang State Polytechnic. The experiments involved different concentrations of CA and C in nine formulations, and the composite resins' mechanical properties and other relevant characteristics were analyzed.

The previous study confirmed the presence of microxyl and amine groups in CA and C, indicating their potential for dental applications [45,46]. Furthermore, the composite resins incorporating these materials demonstrated antibacterial activity against *Streptococcus mutans*, a common oral pathogen, and non-toxic properties, indicating biocompatibility [47].

Secondary caries is typically the cause of restoration failure. The primary cause of this caries is the activity of microorganisms on the tooth's surface. Therefore, developing novel materials capable of eliminating or reducing bacterial acids is imperative [44]. Dental caries is caused by the cariogenic bacterium *Streptococcus mutans*, which forms biofilms and adheres to the surfaces of restorations and teeth. Oral biofilms comprise diverse microbial species established in colonies within a medium composed of salivary proteins, food debris, and microbial components. Caries is caused by acidic byproducts of bacterial metabolism of carbohydrates [38].

The composite resins were divided into nine formulations, each with a different CA and C concentration. Among these formulations, F9, which contained 5 % CA and 7 % C, demonstrated mechanical strength comparable to the control group. F9 showed high absorption (113.33 μg/mm^3^), solubility (80 μg/mm^3^), compressive strength (32.67 MPa), tensile strength (17.18 MPa), and no shrinkage. These findings suggest that F9 has potential as an environmentally friendly dental filling material.

The significance of this study lies in successfully developing a formulation for a restoration material by combining water hyacinth fiber and shrimp shell chitosan. The comparative analysis of the mechanical properties between synthetic composite resins and water hyacinth fiber composites containing shrimp skin chitosan revealed that the F9 formulation (CA 5 % + C 7 %) exhibited favorable properties in terms of absorption, compressive strength, tensile strength, hardness, and thermal expansion.

Using sustainable and biodegradable materials, such as water hyacinth and shrimp shell chitosan, addresses the increasing demand for eco-friendly dental materials [11]. F9 formulation's mechanical properties indicate its potential as a viable alternative to existing dental filling materials. By utilizing waste materials from the seafood processing industry and invasive plant species, this study contributes to environmental sustainability.

However, it is critical to recognize this study's limitations. Mechanical properties are only one aspect of dental restoration materials; additional research is needed to assess other important factors, such as wear resistance, color stability, and long-term durability. In addition, clinical trials and in vivo studies are required to validate the F9 formulation's performance and biocompatibility.

Future research should optimize the formulation by experimenting with different CA and C ratios and concentrations to improve the composite resin's mechanical properties and overall performance. Furthermore, research into the material's long-term stability, degradation, and biocompatibility is critical for its successful implementation in clinical practice.

The current study demonstrates the successful development of an eco-friendly dental restoration material by combining water hyacinth fiber and shrimp skin chitosan. The mechanical properties of the F9 formulation were promising, indicating that it could be a long-term alternative to conventional dental fillings. More research and development are needed to refine the formulation and assess its clinical performance, bringing us closer to developing environmentally friendly and biocompatible dental materials.

## 5 Conclusions

FTIR analysis showed the presence of hydroxyl, primary amine, methylene groups, phenol, functional groups of cellulose acetate, and chitosan. Antibacterial tests using the Flying Paper Disk (FPD) method showed that the CA + C formulation has antibacterial properties. A toxicity test using the BSLT method showed that the CA + C formulation was not toxic. Absorption and solubility in artificial saliva deepening showed that formulation F9 (CA 5 % + C 7 %) had the lowest absorption value while formulation F8 (CA 5 % + C 5 %) had the lowest solubility value. Mechanical properties test results using a Universal Testing Machine (UTM) showed that formulation F9 (CA 5 % + C 7 %) had mechanical strength close to the control group based on absorption, solubility, compressive strength, tensile strength, and no shrinkage. Therefore, the filler formulation and composite resin matrix extracted from water hyacinth and shrimp skin can be an alternative raw material for environmentally friendly dental restorations.

## Statements and declarations

Funding*:* This study was supported by Belmawa, the Ministry of Education, Culture, and Research Republic of Indonesia via the PKM 2023 program, and the Faculty of Dentistry 10.13039/501100010740Hasanuddin University, Indonesia.

## Data availability statement

The data that support the findings of this study are available from the corresponding author (N D) upon reasonable request.

## CRediT authorship contribution statement

**Dewi Nur Fadhila:** Writing – original draft, Validation, Project administration, Methodology, Investigation, Formal analysis, Data curation, Conceptualization. **Alisha Zafirah Ridwan:** Writing – original draft, Project administration, Methodology, Data curation, Conceptualization. **Nur Aqilah Amir:** Project administration, Methodology, Investigation, Data curation. **Andi Abdillah:** Writing – original draft, Investigation, Formal analysis, Data curation. **Ratih Kartini N:** Writing – original draft, Validation, Investigation, Formal analysis, Data curation. **Hasanuddin H:** Writing – review & editing, Writing – original draft. **Nursyamsi Djamaluddin:** Writing – review & editing, Supervision, Project administration, Methodology, Formal analysis, Conceptualization.

## Declaration of competing interest

The authors declare that they have no known competing financial interests or personal relationships that could have appeared to influence the work reported in this paper.
